# Precursor concentration and temperature controlled formation of polyvinyl alcohol-capped CdSe-quantum dots

**DOI:** 10.3762/bjnano.1.14

**Published:** 2010-12-07

**Authors:** Chetan P Shah, Madhabchandra Rath, Manmohan Kumar, Parma N Bajaj

**Affiliations:** 1 Radiation and Photochemistry Division, Bhabha Atomic Research Centre, Trombay, Mumbai - 400 085, India

**Keywords:** cadmium selenide, electron microscopy, energy dispersive X-ray analysis (EDAX), semiconductor

## Abstract

Polyvinyl alcohol-capped CdSe quantum dots, with a size within their quantum confinement limit, were prepared in aqueous solution at room temperature, by a simple and environmentally friendly chemical method. The size of the CdSe quantum dots was found to be dependent on the concentrations of the precursors of cadmium and selenium ions, as well as on the aging time and the reaction temperature; all of which could be used conveniently for tuning the size of the particles, as well as their optical properties. The synthesized quantum dots were characterized by optical absorption spectroscopy, fluorescence spectroscopy, X-ray diffraction, atomic force microscopy and transmission electron microscopy. The samples were fluorescent at room temperature; the green fluorescence was assigned to band edge emission, and the near-infrared fluorescence peaks at about 665 and 865 nm were assigned to shallow and deep trap states emissions, respectively. The quantum dots were fairly stable up to several days.

## Introduction

In recent years, the synthesis and characterization of quantum dots and nanoparticles of group II-VI compound semiconductors have generated a great deal of research interest because of their growing importance in technological applications [1–8], primarily due to the size and the shape dependence of their optical and electrical properties. As a result current research is towards the synthesis and the study of the effect of various experimental parameters on the size of nano semiconductor materials, such as CdSe, with the aim of producing nanoparticles of desired size distribution. Several methods have been employed for the preparation of such nanomaterials [9–18]. One of the most important aspects, in all these methods, is the capping agent, which provides stability to the nanomaterials. Tri-*n*-octylphosphine (TOP), tri-*n*-octylphosphine oxide (TOPO), and aliphatic amines are often used as capping agents. However, the use of such capping agents requires very stringent experimental conditions, such as an inert atmosphere inside a glove box, high temperature etc. [13]. By contrast, the synthesis of nanocrystals, using polymeric stabilizers, is a very easy method, and, in general, only requires ambient laboratory conditions [19–24]. The three-dimensional network of the polymer chains efficiently restricts the growth of these materials to the nanoscale, and the nanomaterials behave like guests in the polymer host. For this purpose, a water-soluble monomer or a polymer compound is generally employed, along with the reagents required for the synthesis of the nanoparticles. Recently, researchers have used a polyvinyl alcohol thin film, to stabilize CdSe quantum dots [25].

In this paper, we report a simple and environmentally friendly method for the synthesis of polyvinyl alcohol-capped CdSe nanoparticles in aqueous medium, under ambient conditions. The effects of the concentrations of the precursors, the aging time and the reaction temperature on the size of the synthesized CdSe quantum dots, as well as the optical absorption and the fluorescence spectra, are presented.

## Results and discussion

**Figure 1:**
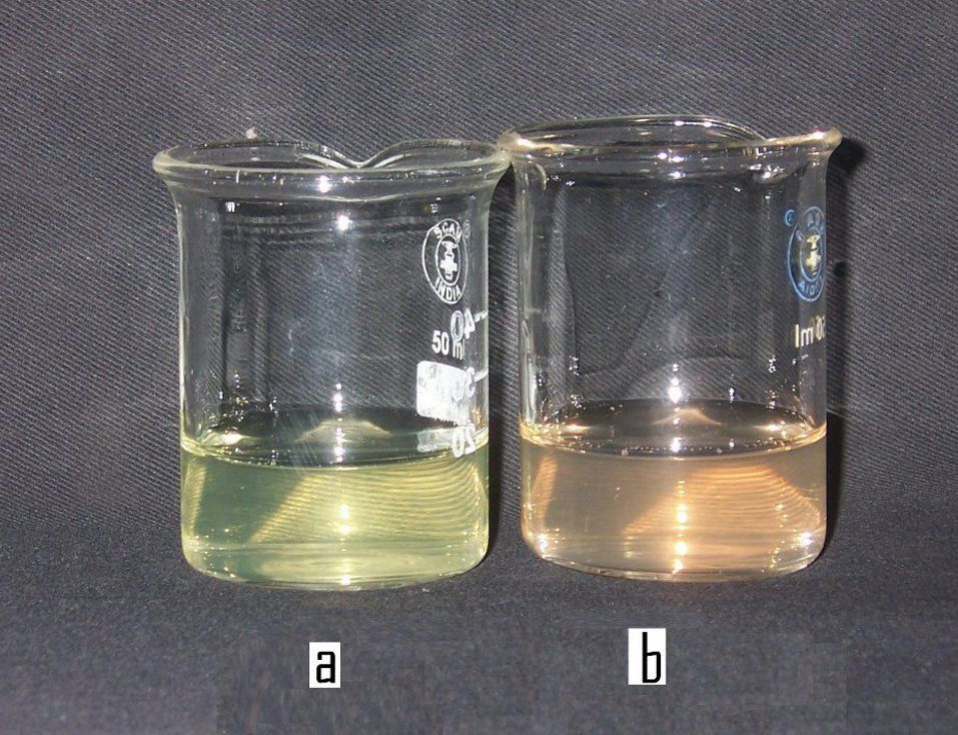
CdSe sols of different colors, synthesized by the reaction of (a) 2 × 10^−3^ mol dm^−3^ Cd(AcO)_2_ and 1 × 10^−3^ mol dm^−3^ Na_2_SeSO_3_, and (b) 2 × 10^−3^ mol dm^−3^ Cd(AcO)_2_ and 4 × 10^−3^ mol dm^−3^ Na_2_SeSO_3_, in the presence of 9.2% PVA, at 27 °C.

CdSe nanoparticles were synthesized by the reaction between Cd(OAc)_2_ and Na_2_SeSO_3_, in the presence of 9.2% PVA, in aqueous medium. Beautiful greenish-yellow, or orange-colored, transparent sols were obtained on stirring the reaction mixture, depending on the concentrations of cadmium and selenium precursors, and the reaction temperature. At room temperature (27 °C), a greenish-yellow colored sol, containing very low sized CdSe particles, was obtained, as shown in Figure 1a, when the CdSe nanoparticles were synthesized by the reaction of 2 × 10^−3^ mol dm^−3^ Cd(OAc)_2_ with 1 × 10^−3^ mol dm^−3^ Na_2_SeSO_3_. Whereas, an orange colored sol of relatively larger CdSe nanoparticles was produced, as shown in Figure 1b, when the CdSe nanoparticles were synthesized by the reaction of 2 × 10^−3^ mol dm^−3^ Cd(OAc)_2_ with 4 × 10^−3^ mol dm^−3^ Na_2_SeSO_3_, irrespective of the reaction temperature. Unless otherwise noted, all the experiments were carried out at room temperature (27 °C). The overall chemical reaction may be represented as follows:


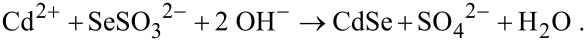


The required alkaline conditions are due to the presence of excess Na_2_SO_3_, which was used in the preparation of a Na_2_SeSO_3_ stock solution. The measured pH of the reaction mixture was ~8, or higher, depending on the concentration of selenium precursor used. Nucleation of CdSe takes place inside the three dimensional network of the PVA chains, which acts as stabilizing agent. The greenish-yellow colored sol did not show any change in color up to about 48 h of aging at room temperature. However, its color slowly changed to light orange, and then to orange, on keeping it for about one week at room temperature, which is probably due to slow aggregation of the initially produced nanoparticles, as monitored by UV–vis spectroscopy.

### Optical absorption study

CdSe sols were synthesized, in the case of greenish-yellow colored sols, by keeping concentration of Cd(OAc)_2_ higher than that of Na_2_SeSO_3_, and vice versa for orange colored sols, as shown in Figure 1a and Figure 1b, respectively.

**Figure 2:**
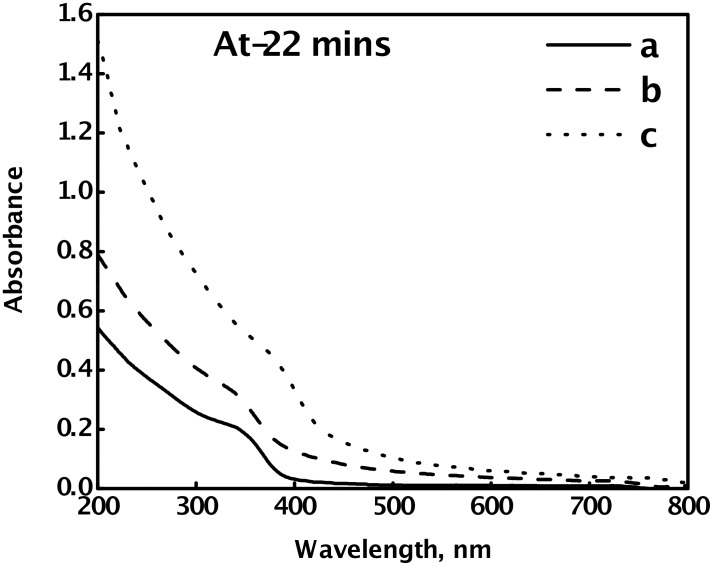
Absorption spectra of PVA-capped CdSe quantum dots, synthesized by the reaction of a fixed Cd(AcO)_2_ concentration of 2 × 10^−3^ mol dm^−3^ with various concentrations of Na_2_SeSO_3_, (a) 4 × 10^−3^ mol dm^−3^, (b) 8 × 10^−3^ mol dm^−3^, and (c) 1 × 10^−2^ mol dm^−3^, in the presence of 9.2% PVA, at 27 °C.

**Figure 3:**
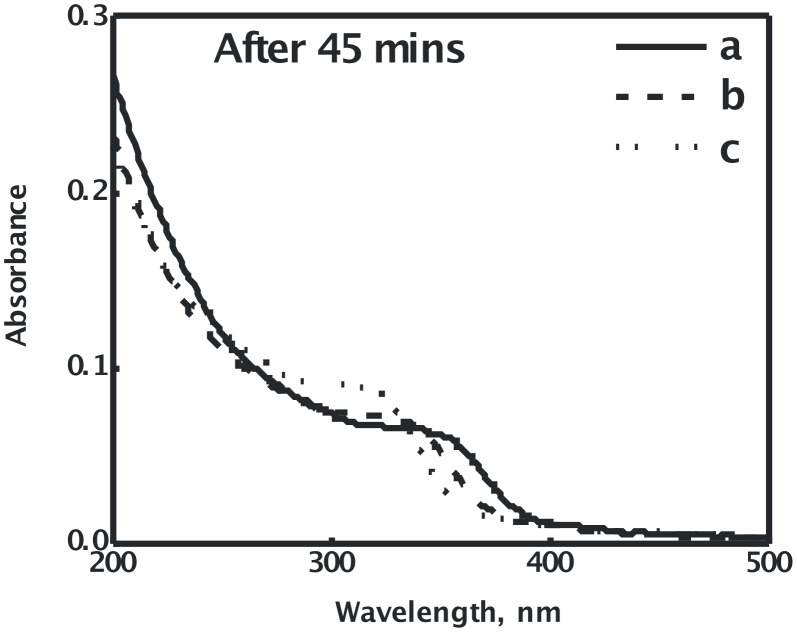
Absorption spectra of PVA-capped CdSe quantum dots, synthesized by the reaction of a fixed Na_2_SeSO_3_ concentration of 1 × 10^−3^ mol dm^−3^ with various concentrations of Cd(AcO)_2_, (a) 2 × 10^−3^ mol dm^−3^, (b) 4 × 10^−3^ mol dm^−3^, and (c) 5 × 10^−3^ mol dm^−3^, in the presence of 9.2% PVA, at 27 °C.

**Figure 4:**
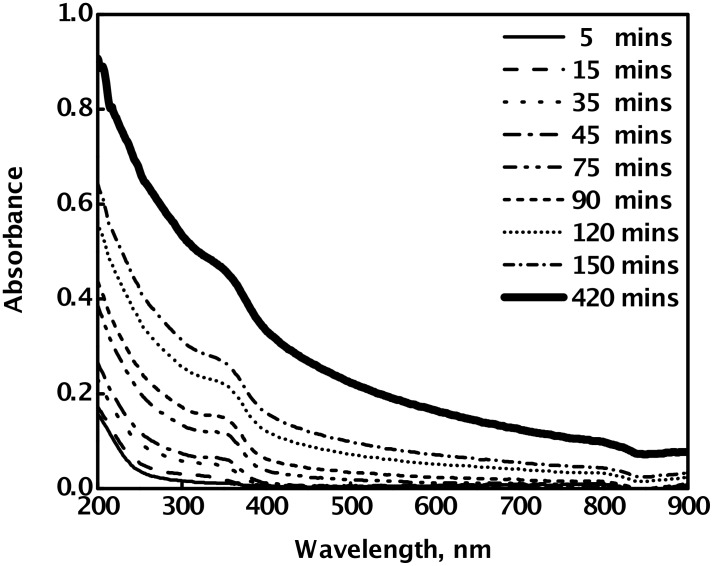
The effect of aging on the absorption spectrum of PVA-capped CdSe quantum dots synthesized by the reaction of 2 × 10^−3^ mol dm^−3^ Cd(AcO)_2_ with 1 × 10^−3^ mol dm^−3^ Na_2_SeSO_3_, in the presence of 9.2% PVA , at 27 °C.

The absorption spectra of aqueous sols, containing CdSe nanoparticles, obtained for various concentrations of the cadmium and the selenium precursors, in the presence of 9.2% PVA, at room temperature (27 °C), are shown in Figure 2, Figure 3 and Figure 4. In all the cases, the absorption edge of the CdSe nanoparticles is significantly blue shifted from the bulk band gap (716 nm), which is a signature of the quantum confinement of the charge carriers in the CdSe nanoparticles. The shift is more pronounced when [Cd(OAc)_2_] > [Na_2_SeSO_3_].

Figure 2 shows the effect of concentration of Na_2_SeSO_3_ on the absorption spectra of the synthesized CdSe nanoparticles, and hence, on the size of the nanoparticles, at a fixed concentration of Cd(OAc)_2_. The excitonic absorption shows a red shift, on decreasing the [Cd(OAc)_2_]/[ Na_2_SeSO_3_] ratio, which indicates an increase in the size of the nanoparticles.

The absorption spectra of CdSe sols, recorded for various concentrations of Cd(OAc)_2_ and a fixed concentration of Na_2_SeSO_3_ (1 × 10^−3^ mol dm^−3^), on maintaining the condition [Cd(OAc)_2_] > [Na_2_SeSO_3_], are shown in Figure 3. A clear excitonic absorption peak is seen in the absorption spectra, which shifts slightly to lower wavelength, on increasing the Cd(OAc)_2_ concentration, which is indicative of a small decrease in the size of the CdSe nanoparticles. Simurda et al. also observed that the size of the CdSe nanoparticles decreased, with an increase in the concentration of cadmium precursor [26].

Figure 4 shows the effect of aging on the absorption spectrum of PVA-capped CdSe sol, synthesized by the reaction of 2 × 10^−3^ mol dm^−3^ Cd(OAc)_2_ with 1 × 10^−3^ mol dm^−3^ Na_2_SeSO_3_. The excitonic absorption peak shifts towards a higher wavelength, as the time progresses, indicating agglomeration to larger CdSe nanoparticles on aging, even in the presence of PVA. It seems that PVA is not able to completely stop the aggregation process, although it reduces its rate drastically. However, in the absence of PVA, agglomeration takes place much faster, leading to almost instant formation of a CdSe precipitate. Further, PVA also assists in restricting the final size of the particles to a certain upper limit, depending on the concentrations of the reagents used. A very similar effect of aging on the optical absorption spectrum of the CdSe nanoparticles was also observed for the other sets of samples, where the concentration of Cd(OAc)_2_ is higher, as well as lower, than the Na_2_SeSO_3_ concentration. When the concentration of the selenium precursor is higher than that of cadmium, the excitonic absorption is red shifted, even at the time of formation of the nanoparticles. It is further red shifted with aging. (Plot is not shown).

In addition to room temperature (27 °C), the reaction was also studied at 10, 22 and 48 °C. The reaction temperature was found to have great influence on the kinetics of formation, as well as the size of the CdSe quantum dots. When the reaction was carried out at 10 °C, with an initial concentration of Cd(OAc)_2_ greater than that of Na_2_SeSO_3_ (2 × 10^−3^ mol dm^−3^ Cd(OAc)_2_ and 1 × 10^−3^ mol dm^−3^ Na_2_SeSO_3_, in the presence of 9.2% PVA), no greenish, or orange color, due to the formation of CdSe nanoparticles, was observed even after ~5 h. Further, when the reaction was carried out at 22 °C, at the same precursor concentrations, still no coloration was seen during the studied time period (~2 h in this case). This is probably due to insufficient release of selenium ions at these temperatures. However, when the reaction was carried out, by keeping the concentration of Na_2_SeSO_3_ higher than that of Cd(OAc)_2_ (2 × 10^−3^ mol dm^−3^ Cd(OAc)_2_ and 4 × 10^−3^ mol dm^−3^ Na_2_SeSO_3_, in the presence of 9.2% PVA), formation of an orange colored CdSe sol was observed at both the lower temperatures, 10 and 22 °C. This is probably due to the increased concentration of selenium ion available at the higher concentration of its precursor. Further, when the reaction was carried out at 48 °C, a lower reaction time of less than 10 min was observed, as compared to that observed at room temperature, under same experimental conditions, irrespective of whether the concentration of cadmium acetate was higher, or lower, than that of sodium selenosulfate. This may be due to the faster release of the selenium ions from its precursor at the higher temperature. The excitonic peak was red shifted on increasing the reaction temperature which is indicative of an increase in the size of the nanoparticles formed.

The size of the CdSe nanoparticles was calculated from the bandgap values, using the Brus equation, which has been simplified to the following equation (Equation 1).

[1]
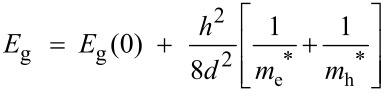


where, *E*_g_ is the bandgap of nanoparticle, *E*_g_(0) is the bulk bandgap, *m*_e_^*^ is the effective mass of the electron and *m*_h_^*^ is the effective mass of the hole, *d* is the size of the nanoparticle, and *h* is the Planck constant.

For CdSe nanoparticles, *m*_e_^*^ and *m*_h_^*^ are 0.13 *m*_0_ and 0.45*m*_0_, respectively [27], and *E*_g_(0) is 1.7 eV [28]. Therefore, the size of CdSe nanoparticles is given by Equation 2.

[2]
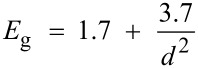


where *d* is in nm.

The bandgap energy was obtained from the absorption spectrum. The sizes of the CdSe nanoparticles determined, using the above equation, for various concentrations of the precursors, by keeping the concentration of Cd(OAc)_2_ fixed, and varying the concentration of Na_2_SeSO_3_, and vice versa, are listed in Table 1. The values of the particles size determined are in agreement with those obtained from the empirical fitting function valid for particle sizes in the range 0.7–8.0 nm [29].

**Table 1:** Particle size of the PVA-capped CdSe quantum dots, synthesized by the reaction of Cd(AcO)_2_ with Na_2_SeSO_3_, in the presence of 9.2% PVA, under the following reaction conditions, as determined from the optical absorption spectra.

Sr. No.	CdSe Sol produced by the reaction of Cd(OAc)_2_ and Na_2_SeSO_3_		

	**[Cd(OAc)_2_] mol dm^−3^**	**[Na_2_SeSO_3_] mol dm^−3^**	**Size (nm)**

**1**	5 × 10^−3^	1 × 10^−3^	1.5 ± 0.2^a^
**2**	2 × 10^−3^	1 × 10^−3^	1.6 ± 0.2^a^
**3**	2 × 10^−3^	4 × 10^−3^	1.6 ± 0.2^b^
**4**	2 × 10^−3^	8 × 10^−3^	1.7 ± 0.3^b^
**5**	2 × 10^−3^	1 × 10^−2^	1.9 ± 0.3^b^

^a^measured after an aging time of 45 min; ^b^measured after an aging time of 22 min.

The particle size was found to be very small, 1.5 nm, when the concentration of Cd(OAc)_2_, 5 × 10^−3^ mol dm^−3^, was higher than that of Na_2_SeSO_3_, 1 × 10^−3^ mol dm^−3^. The size of CdSe quantum dots was found to increase, from 1.6 to 1.9 nm, with an increase in the concentration of Na_2_SeSO_3_, from 4 × 10^−3^ to 1 × 10^−2^ mol dm^−3^, at a fixed 2 × 10^−3^ mol dm^−3^ concentration of Cd(OAc)_2_. The particle size increases from 1.6 nm to 3.3 nm, after aging of the CdSe quantum dots for about 210 h, synthesized by the reaction of 2 × 10^−3^ mol dm^−3^ Cd(OAc)_2_ with 1 × 10^−3^ mol dm^−3^ Na_2_SeSO_3_. Thus, it is clear from the results that, the particle size decreases with an increase in the concentration of Cd(OAc)_2_, or vice versa, and increases with aging time, whether the concentration of Cd(OAc)_2_ is higher, or lower, than that of Na_2_SeSO_3_. Thus, one can achieve selective particle size, by tuning the initial concentrations of Cd(OAc)_2_ and Na_2_SeSO_3_, and the aging time. The quantum confinement limit for CdSe is 5.3 nm [30]. These results clearly indicate that the PVA-capped CdSe nanoparticles prepared in this work, at ambient conditions, are quantum dots. The nanoparticles remain within the quantum-confined limit, even after several days of aging.

The probable reason for the formation of CdSe nanoparticles of smaller size at higher concentration of Cd(OAc)_2_, or vice versa, could be due to the mechanism of nanoparticle formation. Cd^2+^ ions are freely available from the Cd(OAc)_2_ precursor, whereas the counter part is released from SeSO_3_^2−^ much slower. Therefore, the concentration of Cd(OAc)_2_ governs the number of nucleation sites available for the growth of CdSe nanoparticles. For a given concentration of selenium precursor, the greater the number of nucleation sites, the smaller will be the size of the CdSe nanoparticles formed. Similarly, for a given concentration of Cd(OAc)_2_, the number of nucleation sites remains more or less same, and with an increase in the concentration of Na_2_SeSO_3_, the particles size is expected to increase. The reaction temperature has also been found to be a very important factor governing the selenium ion concentration. Thus, the initial concentration of the precursors, the aging time and the reaction temperature can be effectively utilized to produce CdSe quantum dots of the desired size.

### Fluorescence study

**Figure 5:**
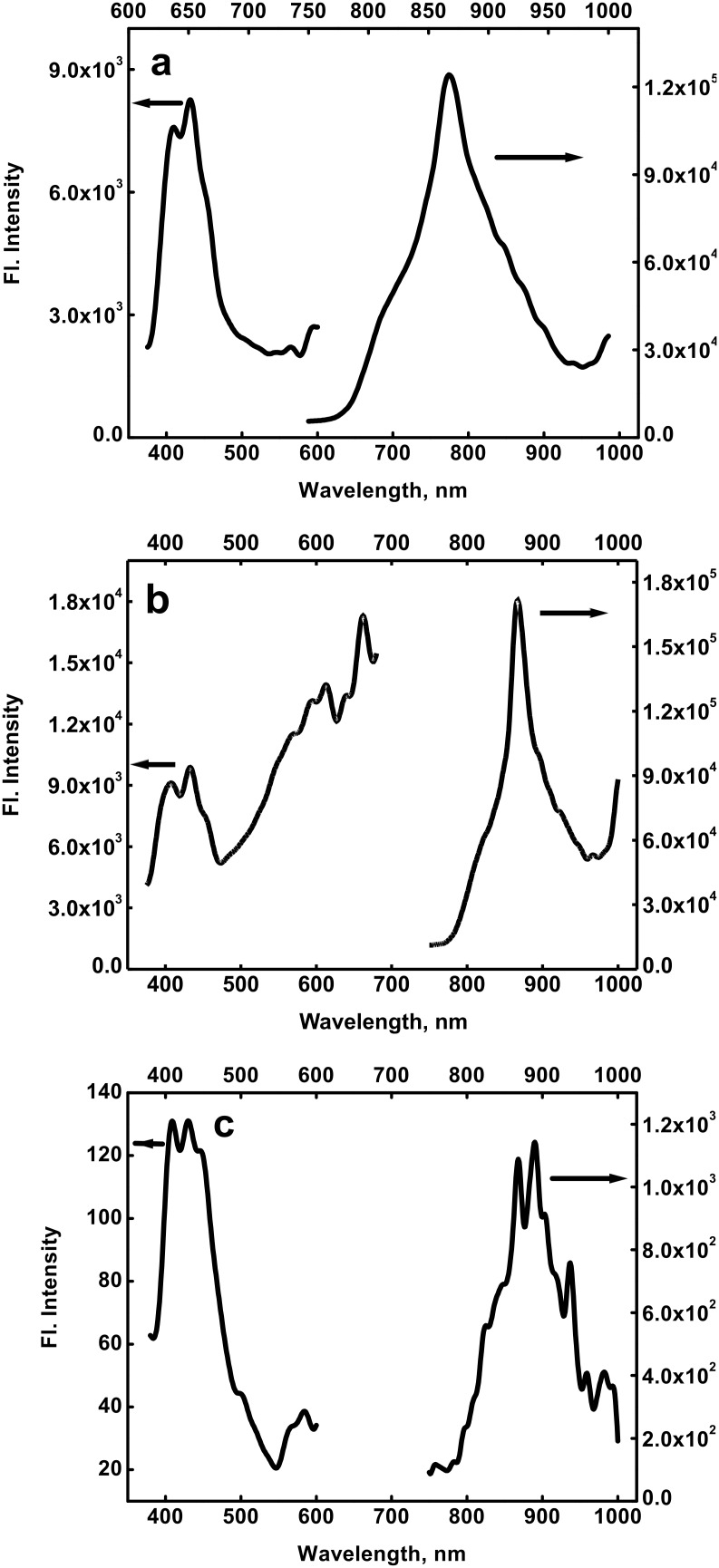
Steady-state fluorescence spectra of PVA-capped CdSe quantum dots, synthesized by the reaction of (a) 2 × 10^−3^ mol dm^−3^ Cd(AcO)_2_ with 1 × 10^−3^ mol dm^−3^ Na_2_SeSO_3_, (b) 4 × 10^−3^ mol dm^−3^ Cd(AcO)_2_ with 1 × 10^−3^ mol dm^−3^ Na_2_SeSO_3_, and (c) 2 × 10^−3^ mol dm^−3^ Cd(AcO)_2_ with 4 × 10^−3^ mol dm^−3^ Na_2_SeSO_3_, in the presence of 9.2% PVA, at 27 °C.

Steady-state fluorescence measurements of the different CdSe sols were carried out at room temperature, by exciting the sample at 350 nm. Fluorescence was observed in all the samples prepared in this study. The fluorescence band, although red shifted with time, is retained for several days, in concurrence with the absorption characteristics. Typical fluorescence spectra of the CdSe sols synthesized at room temperature, with [Cd(OAc)_2_] > [Na_2_SeSO_3_] for two different initial concentrations of Cd(OAc)_2_, 2 × 10^−3^ mol dm^−3^ and 4 × 10^−3^ mol dm^−3^, at a fixed concentration of 1 × 10^−3^ mol dm^−3^ Na_2_SeSO_3_, are shown in Figure 5a and Figure 5b, respectively whilst Figure 5c shows the fluorescence spectrum for [Cd(OAc)_2_] < [Na_2_SeSO_3_], with an initial concentration of 2 × 10^−3^ mol dm^−3^ Cd(OAc)_2_ and 4 × 10^−3^ mol dm^−3^ Na_2_SeSO_3_. All the fluorescence spectra were recorded after one hour of aging.

The visible fluorescence at about 432 nm was observed in all the CdSe quantum dots prepared at various initial concentrations of Cd(OAc)_2_ and Na_2_SeSO_3_, as well as at all the reaction temperatures, and has been assigned to the band edge emission. The near-IR fluorescence at about 865 nm is more intense, as compared to those at about 432 and 665 nm. Moreover, the intensity of the near-IR band increases, as the particle size decreases. The fluorescence peaks at about 665 and 865 nm have been assigned to emission from the shallow trap and deep trap states, respectively. The ratios of the intensities of the band edge emission to those of the trap state emission, after correction for detector spectral sensitivity, are found to be 1:6, 1:13 and 1:3, for the samples a, b, and c, respectively, of Figure 5.

### X-ray diffraction study

**Figure 6:**
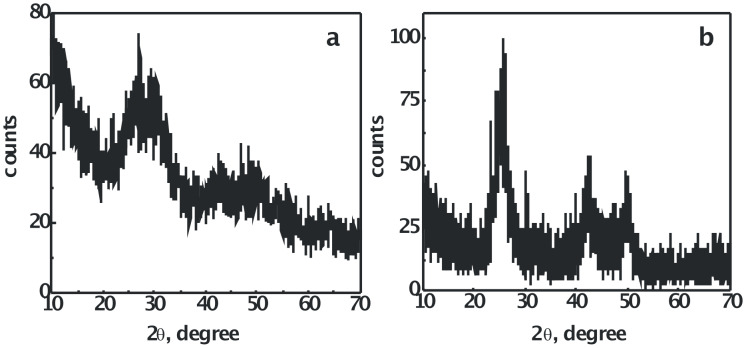
XRD patterns of PVA-capped CdSe quantum dots, synthesized by the reaction of (a) 2 × 10^−3^ mol dm^−3^ Cd(AcO)_2_ with 1 × 10^−3^ mol dm^−3^ Na_2_SeSO_3_, and (b) 2 × 10^−3^ mol dm^−3^ Cd(AcO)_2_ with 8 × 10^−3^ mol dm^−3^ Na_2_SeSO_3_, in the presence of 9.2% PVA, after 48 h of aging. (PVA was removed, and CdSe was washed with water and dried, before XRD analysis).

X-ray diffraction (XRD) patterns of PVA-capped CdSe quantum dots, prepared at two different initial concentrations of Cd(OAc)_2_ and Na_2_SeSO_3_, are shown in Figure 6. The broader nature of the CdSe quantum dots peaks in Figure 6a, where the initial concentration of Cd(OAc)_2_ is higher than that of Na_2_SeSO_3_, as compared to those of the peaks shown in Figure 6b, where the initial concentration of Cd(OAc)_2_ is lower than that of Na_2_SeSO_3_, is in agreement with the trend shown by the optical absorption studies. The observed diffraction peaks of Figure 6b, at 2θ = 25.4°, 42.3° and 49.9°, were assigned to (111), (220) and (311) planes, respectively, of cubic nanocrystalline CdSe. The calculated lattice constant is a = 6.0791 Å, which is in agreement with the literature value of a = 6.077 Å (JCPDS File No. 19-0191).

The size of the nanoparticles *d* was calculated from XRD line broadening data, after instrumental line broadening correction, using Scherrer formula [31], as follows,

[3]
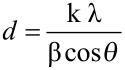


where λ is the wavelength of the X-ray radiation used, and is 0.15406 nm, β is the angular line width at half of the maximum intensity, *θ* is Bragg diffraction angle and k is a constant, and is, in this case, 0.9.

The particle size calculated from Figure 6b, for the sample prepared, with the concentration of Cd(OAc)_2_ lower than that of Na_2_SeSO_3_, was found to be 2.4 nm. The calculation of particle size for the sample, synthesized from the initial concentration of Cd(OAc)_2_ higher than that of Na_2_SeSO_3_, could not be carried out, since it did not prove possible to obtain the β-value from the XRD pattern.

Further, the particle size was also calculated, using the Williamson–Hall equation [32]. By plotting the value of β cos *θ* against 4 sin *θ*, the lattice strain of the nanocrystal *ε* can be obtained from the slope, and the crystallite size from the intercept on the vertical axis, as per Equation 4.

[4]
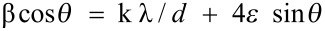


**Figure 7:**
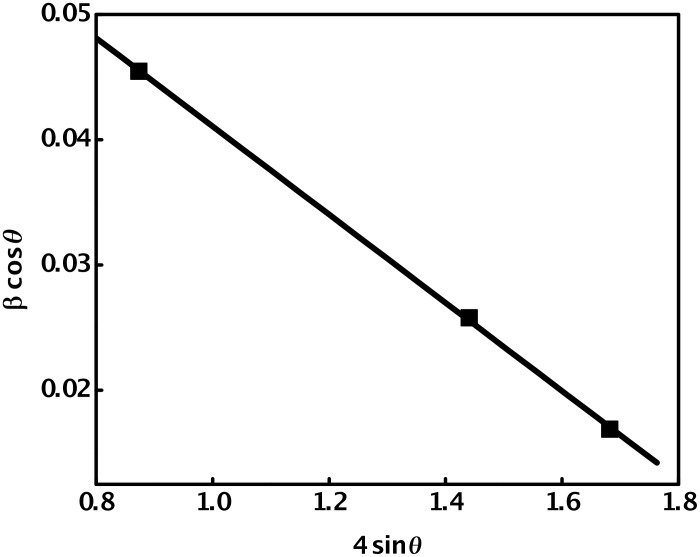
Williamson–Hall plot of PVA-capped CdSe quantum dots, synthesized by the reaction of 2 × 10^−3^ mol dm^−3^ Cd(AcO)_2_ with 8 × 10^−3^ mol dm^−3^ Na_2_SeSO_3_, in the presence of 9.2% PVA, after 48 h of aging.

Figure 7 shows the Williamson–Hall plot of CdSe quantum dots, synthesized by the reaction of 2 × 10^−3^ mol dm^−3^ Cd(AcO)_2_ with 8 × 10^−3^ mol dm^−3^ Na_2_SeSO_3_, in the presence of 9.2% PVA, after 48 h of aging. The negative slope of the plot indicates that strain broadening must be very small [33]. The particle size was found to be 1.7 nm from the intercept. The particle size found by the three methods, UV−vis spectroscopy, Scherrer equation and Williamson–Hall equation, are quite close to one another.

**Figure 8:**
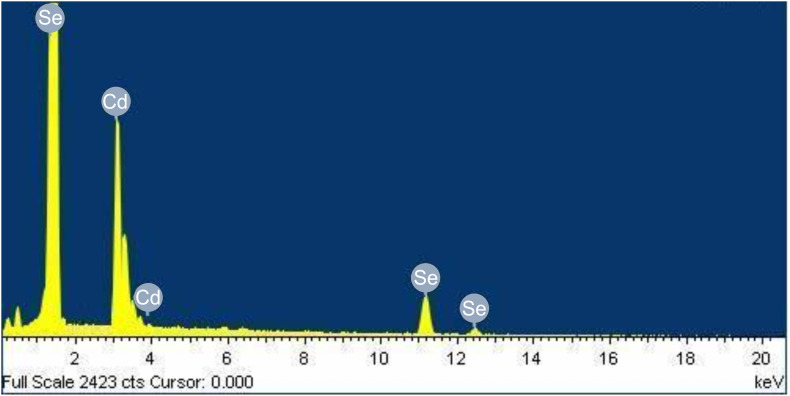
EDAX pattern of PVA-capped CdSe quantum dots synthesized by the reaction of 2 × 10^−3^ mol dm^−3^ Cd(AcO)_2_ with 4 × 10^−3^ mol dm^−3^ Na_2_SeSO_3_, in the presence of 9.2% PVA, at 48 °C.

Further, the chemical composition of the synthesized cadmium selenide quantum dots was also confirmed by EDAX, as shown in Figure 8. It indicates the presence of cadmium and selenium, along with very small amounts of carbon and oxygen, probably due to incomplete removal of PVA. The areas of the peaks, after correction for the X-ray emission cross-section of Cd and Se, indicate the stoichiometry of the cadmium selenide quantum dots formed as 1:1, i.e., Cd_1_Se_1_.

### TEM and AFM studies

**Figure 9:**
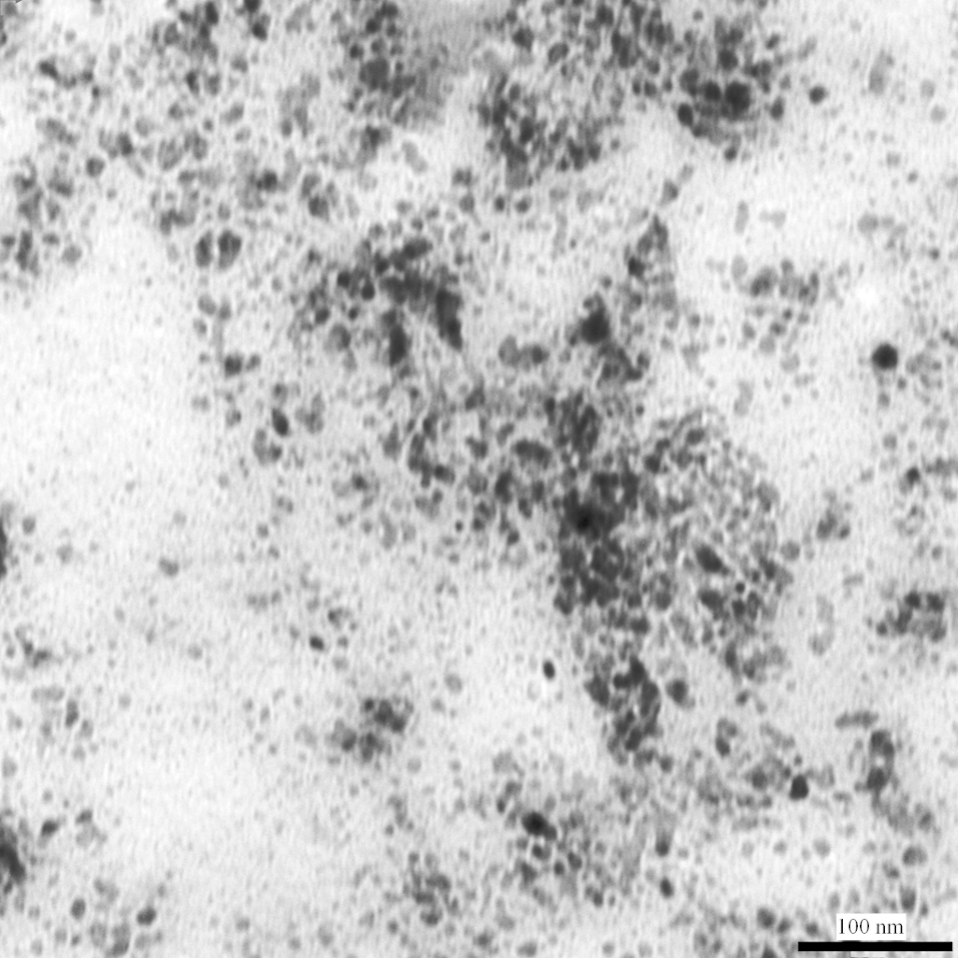
Typical TEM image of the CdSe quantum dots, synthesized by the reaction of 2.0 × 10^−3^ mol dm^−3^ Cd(AcO)_2_ with 1.0 × 10^−3^ mol dm^−3^ Na_2_SeSO_3_, in the presence of 9.2% PVA, at 27 °C. TEM image was taken 6 months after the synthesis of the CdSe nanoparticles.

Transmission electron microscopy and atomic force microscopy are very important techniques for providing information about particle size, shape, surface topography, etc. Thus, the morphology and structure of the synthesized CdSe quantum dots were also investigated by TEM and AFM techniques. A typical TEM image of CdSe quantum dots, synthesized by the reaction of 2.0 × 10^−3^ mol dm^−3^ Cd(AcO)_2_ with 1.0 × 10^−3^ mol dm^−3^ Na_2_SeSO_3_, at 27 °C, in the presence of 9.2% PVA, is shown in Figure 9.

The particles were found to be spherical agglomerates, with an average size of ~10 nm. A careful analysis revealed that, probably, these spherical agglomerates were composed of small CdSe nanocrystals, which have much smaller dimensions. Both small and large aggregates can be seen in Figure 9. TEM measurements could not be carried out in the presence of PVA, because of charging of the sample due to its non-conducting nature. Therefore, PVA was removed, before loading the sample on the copper grid, which led to aggregation of the individual nanoparticles during drying of the sample.

**Figure 10:**
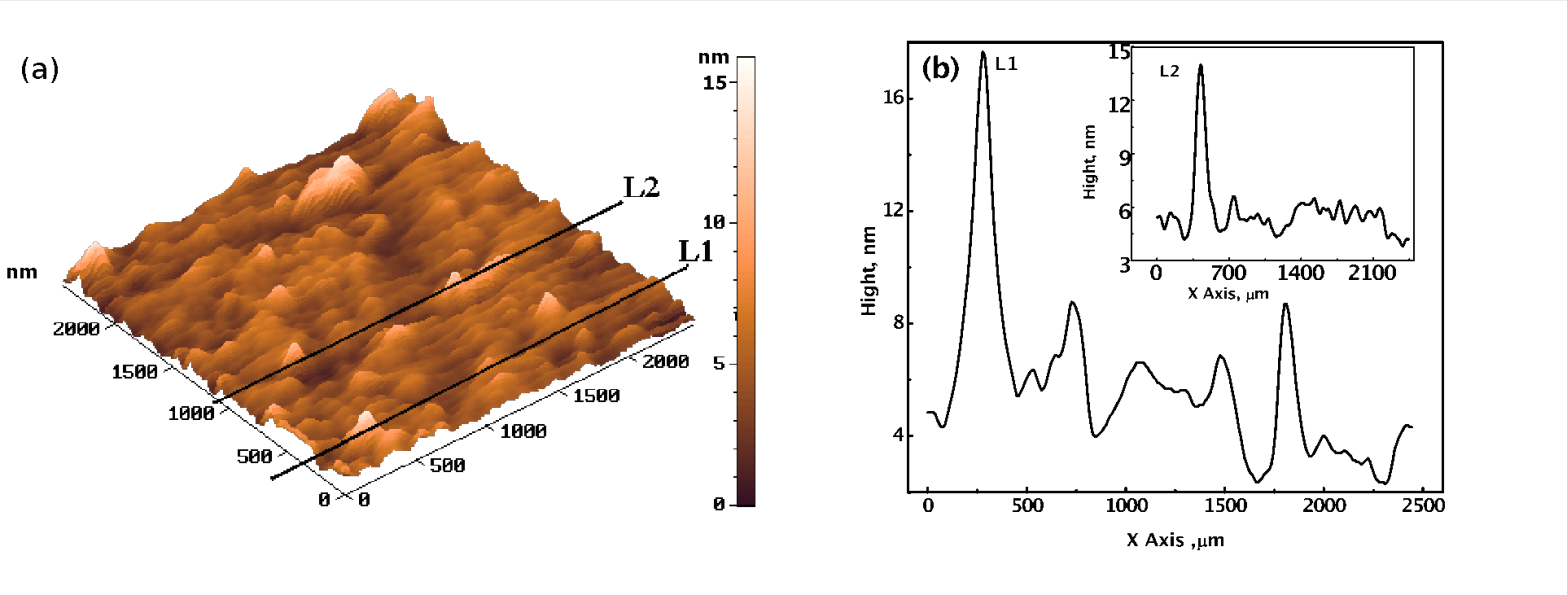
AFM image of the CdSe quantum dots, synthesized by the reaction of 2.0 × 10^−3^ mol dm^−3^ Cd(AcO)_2_ with 1.0 × 10^−3^ mol dm^−3^ Na_2_SeSO_3_, in the presence of 9.2% PVA, at 27 °C, (a) 3D image, and (b) height profiles of lines L1 and L2.

Further, typical 3D AFM image of the CdSe nanoparticles is shown in Figure 10. The Z-direction profiles of the lines L1 and L2 of the 3D AFM image are also shown (Figure 10b). It indicates the presence of nanoparticles/aggregates, with a height in the range of 6 to 13 nm. The background height variation of ~5 nm is also quite evident from the 3D image (Figure 10a). The size resolution limit of the employed AFM machine is ~5 nm. Under the mentioned instrumental limitations, it can be concluded from both the TEM and AFM images that, the CdSe nanoparticles/aggregates of dimensions less than ~10 nm are formed.

The results from UV−vis spectroscopy, fluorescence measurements and XRD clearly indicate the formation of individual CdSe nanoparticles, with sizes within the quantum confinement limit.

## Conclusion

In summary, PVA-capped CdSe quantum dots were synthesized at room temperature, by a simple chemical method. These exhibited excitonic absorption as well as emission patterns. The size of the CdSe nanoparticles could be controlled, by varying the initial concentrations of the precursors of cadmium and selenium ions, aging time and the reaction temperature. Greenish-yellow colored CdSe sols, containing very small size quantum dots, were obtained, when the concentration of Cd(AcO)_2_ was higher than that of Na_2_SeSO_3_, at room temperature, and orange-colored CdSe sols, containing relatively larger quantum dots, were obtained, when the concentration of Na_2_SeSO_3_ was higher than that of Cd(AcO)_2_, in the studied temperature range of 10° to 48 °C. The CdSe quantum dots were fairly stable up to several days. Particle size analysis, using different techniques, confirmed that, the size is within the quantum confinement limit. Thus, this method provides an easy route for preparation of luminescent PVA-capped CdSe quantum dots of very small size, without the use of harsh experimental conditions, generally employed in the reported methods.

## Experimental

Polyvinyl alcohol (PVA) (molecular weight 125,000) and cadmium acetate (Cd(OAc)_2_) were obtained from S.D.Fine-Chem Ltd., Mumbai, India, and were used as received. All the other chemicals used were of A.R. grade. Solutions were prepared, in water obtained from Millipore-Q water purification system (with conductivity of 0.6 µS cm^−1^ or lower). Sodium selenosulfate (Na_2_SeSO_3_) was prepared by the reported method, from Na_2_SO_3_ and Se powder [34]:


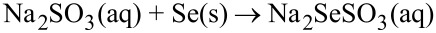


Briefly, a mixture of selenium powder (1 g) and Na_2_SO_3_ (10 g), in 50 mL water was heated under reflux in a 100 mL round-bottomed flask at 70 °C for about 7 h. After completion of the refluxing process, the reaction mixture was filtered and the clear transparent solution obtained was kept in dark to prevent photo-oxidation. The yield of sodium selenosulfate was 99%, based on the amount of selenium powder consumed in the reaction. The Na_2_SeSO_3_ solution (0.25 M) thus prepared was used as a stock solution for the Se source. Cd(OAc)_2_ solution (0.5 M) was used as a stock solution for the Cd source. A stock solution of PVA was prepared by adding 10 g PVA powder to 100 mL water with constant stirring at 80 °C, to yield a transparent viscous solution. The above stock solutions were further diluted to the required concentrations for different experiments.

PVA-capped CdSe nanoparticles were synthesized by mixing measured volumes of the aqueous solutions of the precursors, Cd(OAc)_2_ and Na_2_SeSO_3_, of various concentrations, in the presence of the required concentration of PVA, and stirring the mixture constantly at different temperatures until completion of the reaction. The reaction was carried out in a jacketed container, and temperature control of the reaction mixture was maintained by circulating water from a thermostated bath, with temperature variation less than ±0.2 °C. Most of the experiments were carried out at room temperature, and the reaction was complete within 30 min.

Absorption and fluorescence spectra of the synthesized CdSe nanoparticles were recorded, using Chemito Spectroscan UV-2600 spectrophotometer and an Edinburgh Instruments, Ltd FLSP920 system with 450 W Xenon lamp. XRD patterns were recorded on a Phillips PW 1710 X-ray diffractometer, using Cu Kα radiation (λ = 0.15406 nm). Transmission electron microscopy (TEM) characterization was carried out on a Libra-120 electron microscope, by loading the sample on a copper grid coated with a thin amorphous carbon film. AFM analysis of the cadmium selenide nanoparticles was carried out with Solver P47 model from NT-MDT, Russia, by loading the sample on a polished silicon wafer. The presence of PVA in the aqueous CdSe sols interfered in TEM and AFM imaging. Therefore, CdSe nanoparticles were separated from the aqueous sols using a high-speed centrifuge, washed and re-dispersed in water using an ultrasonicator before preparing samples for TEM and AFM analyses.
